# A Review of Topical Phage Therapy for Chronically Infected Wounds and Preparations for a Randomized Adaptive Clinical Trial Evaluating Topical Phage Therapy in Chronically Infected Diabetic Foot Ulcers

**DOI:** 10.3390/antibiotics9070377

**Published:** 2020-07-04

**Authors:** Christopher Anthony Duplessis, Biswajit Biswas

**Affiliations:** Naval Medical Research Center, 503 Robert Grant Avenue, Silver Spring, MD 20910, USA; Biswajit.biswas.civ@mail.mil

**Keywords:** bacteriophage, wounds, ulcers, topical, infected ulcers, infected wounds, topical therapeutics, phage library, phage-antibiotic synergy, phage-phage synergy, and antibiotics

## Abstract

The advent and increasing prevalence of antimicrobial resistance commensurate with the absence of novel antibiotics on the horizon raises the specter of untreatable infections. Phages have been safely administered to thousands of patients exhibiting signals of efficacy in many experiencing infections refractory to antecedent antibiotics. Topical phage therapy may represent a convenient and efficacious treatment modality for chronic refractory infected cutaneous wounds spanning all classifications including venous stasis, burn-mediated, and diabetic ulcers. We will initially provide results from a systematic literature review of topical phage therapy used clinically in refractorily infected chronic wounds. We will then segue into a synopsis of the preparations for a forthcoming phase II a randomized placebo-controlled clinical trial assessing the therapeutic efficacy exploiting adjunctive personalized phage administration, delivered topically, intravenously (IV) and via a combination of both modalities (IV + topical) in the treatment of infected diabetic foot ulcers (perhaps the canonical paradigm representing complicated recalcitrant infected cutaneous wounds).

## 1. Introduction

Antimicrobial resistance saliently multi-drug-resistant (MDR) and extensively drug-resistant (XDR) bacterial infections] increasingly engenders infections refractory to treatment establishing a public health crisis. Most alarming, there are few novel antibiotics appearing in the development pipeline. The World Health Organization (WHO) has forecast a ‘pre-antibiotic era’ in its 2014 surveillance report [1]. The death toll attributed to MDR organisms is estimated to eclipse 23,000 in the U.S., and half a million people globally annually [2,3,4], and may reach 10 million in 2050 at the current trajectory [4]. The health care costs attributed to, and resources expended on antibiotic resistance is staggering. The estimated annual costs are 1.5 billion euros in Europe [1], $200 million in Canada, and up to $77.7 billion in the USA [2,3,4].

Concerted efforts are required to identify non-antibiotic antimicrobials to treat MDR bacterial infections. One promising option includes bacteriophage (phage) therapy, which has been resurrected as a viable clinical therapeutic. Phages are viruses that infect and replicate within bacteria eventually killing their prokaryotic host (but do not replicate in eukaryotic cells). Therefore, phages are intrinsically safe in humans as they are exquisitely specific in targeting and infecting bacteria to the species and often the strain level. Myriad observational studies (predominantly borne from the Eastern bloc nations and Russia) universally confirm phage safety when exploited for clinical therapy. Furthermore, phages comprise an appreciable (largest) percentage of the human microbiome, and are ubiquitously present throughout our environment including food and water sources. There are an estimated 10^31–32^ phages on the planet thus representing the most abundant and diverse life form in the biosphere. Phages were used for treating infectious diseases in the 1930s; however, enthusiasm was curtailed, and research mostly abandoned in the 1940s due to the advent of antibiotics [2,3,4,5,6]. At the forefront of this phage renaissance, are two centers for phage research and clinical therapeutics, which have remained active since the inception of phage use for clinical treatments: (1) The Eliava Institute and the Eliava Phage Therapy Center in the Republic of Georgia, and (2) the Institute of Immunology and Experimental Therapy, Polish Academy of Sciences, in Wroclaw, Poland. 

Bacteriophages exhibit numerous potential advantages as an antimicrobial for adjunctive therapy for treatment of MDR bacterial infections including: (1) clinical safety; (2) bactericidal vice bacteriostatic activity (irrespective of antibacterial resistance profiles); (3) increased concentration where desired (at the site of infection); (4) reduced collateral damage to the microbiome (and given the specificity lack of engendering non-targeted bacterial resistance); (5) potential in vivo antibiotic synergy; (6) potential reversion of bacterial antibiotic susceptibility in vivo; (7) biofilm degrading activity; (8) anticipated cost-effectiveness of pharmaceutical development; (9) ease and rapidity in isolation, and (10) amenability to engineering [4,5,6,7,8,9,10,11,12,13]. Of import, the resources required to develop novel phage therapeutics is vastly cheaper than that of antibiotics and other antimicrobials. Potential disadvantages associated with phages include: (1) unfamiliarity and resistance to its widespread adoption and implementation by the public; (2) phage specificity requiring precision identification of phages targeting the exact infection (requires time and resources); (3) allergic responses (although infrequently observed in clinical practice); (4) bacterial resistance development during treatment, and (5) lack of evidence-based guidance governing treatment regimens regarding optimal (dosing, and frequency, duration and route of administration) [4,5,6,7,8,9,10,11,12,13]. With dedicated methodologically rigorous research devoted to evaluating phage efficacy in the clinical arena, many of the aforementioned disadvantages will likely be mitigated if not eliminated. 

In fact, numerous commercial products encompassing well characterized phages (phage cocktails or phage mixtures) are currently marketed for clinical, veterinarian, and environmental applications. Many commercial products are marketed to cater to specific clinically germane syndromic infections, and encompass phages targeting the most common associated pathogenic species implicated based upon contemporary epidemiology. Some salient representative examples of commercial phage products currently marketed for the treatment of cutaneous infections include, (a). PhagoBioDerm (from Intralytix, a product originally licensed in the Republic of Georgia), and ((b) Phagoderm (from Micro World in Russia). In similar fashion, for treating purulent infections there are: (a) PYO Bacteriophage (from Eliava BioPreparation); and ((b) Septaphage (from Biochimpharm in the Republic of Georgia). For the treatment of diarrheal infections there are: (a) Intesti Bacteriophage (from Eliava BioPreparation); ((b) Dysentery Bacteriophage (from Microgen in Russia); (c) Travelphage (from Biochimpharm in the Republic of Georgia); (d) Intesti Bacteriophage (from Microgen in Russia); and (e) Intestifag (from NeoProbioCare in Ukraine). Finally, there are well characterized commercially available fixed phage mixtures formulated for promotion of food safety derived from Intralytix (in the U.S.) including: (a) ListShield (targeting *Listeria monocytogenes*); ((b) EcoShield (targeting *Escherichia coli*); (c) SalmoFresh (targeting highly pathogenic *Salmonella*-serotypes); and (d) ShigaShield (targeting three major *Shigella* species; *S. flexneri*, *S. sonnei* and *S. dysenteriae*.

Numerous observational studies confirm the safety of bacteriophage when employed clinically, while exhibiting signals of therapeutic efficacy. Methodologically rigorous clinical trials are now warranted to confirm results acquired from observational studies and clarify the potential role of phage in our therapeutic armamentarium for treatment of bacterial infections. Bacteriophage therapy has rapidly become a highly sought emergency treatment [administered under Emergency Investigational New Drug Applications (eINDs)] for refractory MDR bacterial infections. A recent case series reporting upon the compassionate use eINDs cataloguing adjunctive personalized phage therapy for patients experiencing refractory MDR bacterial infections identified safety, and signals of clinical efficacy reflected by confirmed microbiological eradication, and clinical improvement (survival) [14]. 

Chronically infected cutaneous wounds (ulcers) including infected diabetic ulcers are often refractory to systemic antibiotic treatment due to (a). poor vascularity; ((b). biofilm activity (circumventing penetration), and (c). bacterial resistance, thus swiftly becoming a public health crisis. Therefore, adjunctive phage therapy may be warranted for treating these refractory infections. Intuitively, phages may be best administered topically, securing direct access to the infection, while remaining shielded from host systemic immunity. The topical (relative to intravenous (IV)) administration may serve as a safer, more cost-effective, and most convenient route for phage delivery, amenable to direct instillation, impregnation in novel dressings, or encapsulation in varied vehicles (i.e., hydrogels). However, IV phage administration may be required for systemic infections and access to sequestered bacteria in deep tissue recesses inaccessible by isolated topical delivery.

While pursuing a randomized clinical trial to assess the safety and efficacy of adjunctive topical and non-topical (IV) phage administration for the treatment of recalcitrant infected diabetic foot infections, we completed a systematic literature review to ascertain the clinical experience achieved with phages administered topically to patients experiencing any recalcitrant chronically infected cutaneous ulcer including (diabetic, venous stasis, decubitus, and burn-mediated ulcers). Thereafter, we present a synopsis of our unique preparations for executing the clinical trial. 

## 2. A Systematic Review of Topical Bacteriophage Therapy Used in Chronically Infected Ulcers 

### 2.1. The Literature Search

We searched electronic databases (Medline, Embase, and Cochrane database of clinical trials) from 1980 to April, 2020 using the following terms: “*phage and infected wound”, “*phage and infected ulcer”, “*phage and diabetic”, “*phage and venous”, “*phage and decubitus”, “*phage and pressure ulcer”, “*phage and topical”, “*phage and cutaneous”, and “*phage and burn”. Note, the asterisk represents a wildcard character ensuring capture of “phage” and “bacteriophage” within the search terminology. The broad “umbrella” terminology employed potentiated casting of a wide net to capture an exhaustive publication list. The reference sections from evaluated articles were reviewed for satisfying the inclusion criteria.

### 2.2. Inclusion/Exclusion Criteria

#### 2.2.1. Inclusion Criteria

All articles were reviewed for eligibility criteria. To be included, studies were required to: (1) Report upon human trials (any age) assessing chronic recalcitrant wound infections conforming to any of the accepted search terms encompassing infected diabetic, venous stasis, decubitus (pressure ulcer), or burn associated wounds/ulcers. Accepted articles could additionally report agnostically upon any infected, purulent, or suppurative cutaneous wound (ulcer), (2) Publish in the English language between the years 1980–April, 2020, (3) Report results regarding evaluated safety monitoring, microbiological surveillance, or clinical endpoints (i.e., wound healing assessments), (4) Administer topical phage as adjunctive therapy. (a). Concomitant antibiotics, alternative therapeutic regimens, and alternative routes of phage delivery (oral and/or IV administration) were acceptable if the non-topical phage delivery was intended solely to contribute to the treatment of the infected cutaneous wound/ulcer. (b). There were no constraints imposed upon topical phage delivery methods (i.e., methods for impregnation in dressings or encapsulation in hydrogels). (c). There were no constraints imposed upon phage administration regimens (i.e., no dictating of dosing, frequency, or duration), acknowledging anticipated heterogeneity absent current guidelines governing optimal therapeutic regimens.

#### 2.2.2. Exclusion Criteria

**Figure 1:**
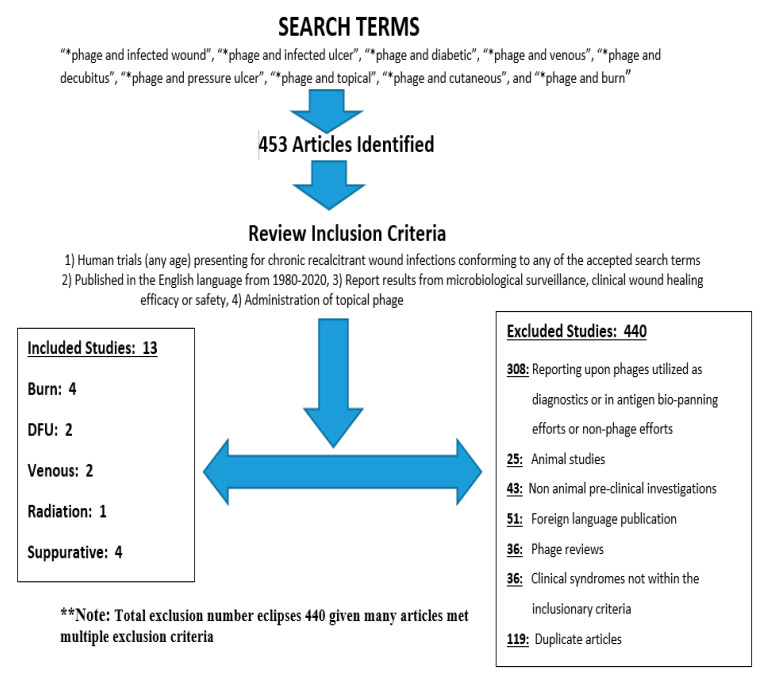
Flow chart depicting search methodology^1^. (1). Report upon human trials (any age) assessing chronic recalcitrant wound infections conforming to any of the accepted search terms encompassing infected diabetic, venous stasis, decubitus (pressure ulcer), or burn associated wounds/ulcers. Accepted articles could additionally report agnostically upon any infected, purulent, or suppurative cutaneous wounds/ulcers. (2). Publication is in the English language between the years 1980–2020. (3). Report provides results from microbiological surveillance data and clinical wound healing efficacy. (4). Interventions reported must include the administration of topical phage: (a). concomitant antibiotics and alternative modes of phage delivery (oral and/or IV delivery) are acceptable; ((b). there were no constraints imposed upon the delivery vehicle, or administration regimen [dosing, or administration (frequency or duration)] ^2^Total exclusion criteria met eclipses 440 as many articles met multiple exclusion criteria.

Exclusionary criteria included studies failing to adequately report results regarding safety, or microbiological and clinical assessments thought attributed to introduction of topically delivered phage therapy for chronically infected cutaneous ulcers as described in the inclusion criteria. Specific a-priori exclusion criteria included: (a). post-surgical infected wounds; ((b). mucosal infections (i.e., rhinosinusitis); (c). otitis externa; (d). pleuro-pulmonary infections, and (e). gastrointestinal (GI), genitourinary (i.e., cystitis), and recto-vaginal (applied via tampons or pessaries) infections. Figure 1 presents the flow chart delineating the search methodology and results.

### 2.3. Data Abstraction

The following data were abstracted (if available), and entered into a MS Excel^®^ worksheet for analysis: author, publication year, number of patients treated, microbiology (infecting bacteria targeted), employed phage(s) or phage cocktails cataloguing their bacterial specificity, and whether fixed or personalized (confirmed to lyse the patient’s infecting bacteria isolate in vitro), phage therapeutic regimen employed (dosing, frequency and duration of administration), ancillary treatments (i.e., antibiotics, non-antibiotic rheological agents), and microbiological and clinical efficacy endpoints attributed to phage therapy. Additionally, exclusionary criteria were documented (animal study, non-animal pre-clinical study, non-phage investigation, non-clinical phage investigation, foreign language publication, phage review, and clinically mediated phage therapy not meeting the inclusion criteria. 

### 2.4. Analysis

The main clinical endpoints to be culled from the included studies are (1) safety assessments, (2) microbiological assessments concentrating on reductions in bacterial burden post-phage treatments, and (3) the efficacy in wound healing operationalized as rates of complete wound recovery or parameters indicating wound healing including (reductions in purulence and/or wound size and depth, or wound granulation or epithelialization) thought attributable to topical phage therapy. The overall efficacy culled from all included studies will be captured as point estimates along with standard 95% confidence intervals and estimated using a random-effects model (DerSimonian & Laird, 1986). Heterogeneity in study wound healing rates will be assessed using a *χ*^2^ statistic, and graphically represented with Forest plots. Statistical analyses will be performed with Stata Version 10 (StataCorp. College Station, TX, USA).

## 3. Literature Search Results

### 3.1. Overall Search Results

Our search resulted in 453 articles from which we identified 13 studies meeting the inclusion criteria (440 articles excluded). Most excluded articles (308) were due to reporting upon phages used in a diagnostic capacity (i.e., antigen biopanning efforts), or investigations not involving phage therapy in any capacity (pre-clinical or clinical). Additional exclusionary criteria included animal studies (25), non-animal pre-clinical investigations (43), foreign language publication (51), phage reviews (36), clinical phage therapy exploited in syndromes not conforming to the inclusionary criteria (34), and duplicate articles (119). Note, many articles fulfilled multiple exclusionary criteria hence numbers eclipse the total article sum.

### 3.2. Synopses of the Results from Included Articles (See Table 1)

#### 3.2.1. Topical Phage Therapy for Treatment of Infected Venous Stasis Wounds/Ulcers

Successful topical phage treatment was achieved in 67 of 96 (70%) patients afflicted by infected venous stasis ulcers, or a heterogeneous assortment of infected “purulent” ulcers refractory to antecedent antibiotic treatment observed from 1999–2000 in Tbilisi, Georgia (Table 1). Clinical improvement in wound healing was observed in another 24 patients (defined by reduced ulcer size, and elimination of purulent drainage). The treatment duration extended between 6 days to 15 months. The phage therapy was administered via a commercial dressing “PhagoBioDerm” licensed in the Republic of Georgia in 1999 to treat chronically infected wounds. The PhagoBioDerm product is a novel biodegradable polymer impregnated with the following therapeutics: (a). lytic bacteriophages (the characterized “pyophage cocktail”) encompassing up to 10^6^ plaque-forming units (pfu) of *Pseudomonas aeruginosa (PsA)*, *Escherichia coli (E. coli)*, *Staphylococcus aureus (S. aureus)*, *Streptococcus* spp., and *Proteus* spp. targeting phages; (b). antibiotics (ciprofloxacin 0.6 mg); (c). anesthetic (benzocaine 0.9 mg), and (d). a wound healing agent (α-chymotrypsin) [15]. The product is reapplied as needed (prn) upon degradation of the polymer and continued need for wound healing. Of 22 cases in which microbiologic data were available, healing was associated with concomitant elimination of the infecting pathogen [15]. The authors acknowledge limitations including the absence of a control group, and concomitant wound healing therapeutics applied. However, these highly selected patients failed antecedent standard of care therapy including antibiotics, and exhibited remarkable improvement upon initiation of topical therapy with the PhagoBioDerm product.

A safety evaluation exploiting topical phage therapy [employing a fixed 8-phage cocktail targeting *Pseudomonas* spp., *E. coli*, and *S. aureus*—“WPP-201” from Intralytix] was undertaken in 39 subjects (18 subjects received phage therapy, 21 controls) exhibiting chronic venous leg ulcers, (infected or uninfected) in a formal phase I randomized double-blind placebo-controlled (saline) investigation conducted in Texas, U.S. in (2007–2008). The phage was applied via impregnated dressings weekly for 12 weeks and follow-up occurred at 24 weeks [16]. This trial is considered the first phase I trial exploiting phage therapy in the U.S. executed under NCT 00663091. Although this was designed solely as a safety evaluation (confirmed), wound epithelialization was achieved in 17/21 control patients and 12/18 treated patients at week 24 (non-significant). No interpretations may be levied regarding microbiological outcomes as cultures were not acquired. Interpretations related to clinical efficacy are limited given that bacterial sensitivities to the phage constituents were not executed. 

#### 3.2.2. Topical Phage Therapy for Secondarily Infected Radiation Induced Cutaneous Ulcers

A case series reported upon the successful application of PhagoBioDerm therapy applied to refractory secondarily infected (*S. aureus*) radiation induced ulcers [stemming from complications of strontium-90 induced injury] in two lumberjacks in 2002. After failing one-month of antibiotic therapy, a single application of PhagoBioDerm treatment (executed in Tbilisi, Georgia) yielded microbiological eradication, reduced purulent drainage, and promotion of clinical healing ensuing over a course of 2–7 days [17]. Of note, and not a subtle observation, the authors confirmed that the infecting *S. aureus* isolates acquired from both patients were sensitive to the phages, and resistant to ciprofloxaxin represented within the composition of the PhagoBioDerm product [17].

#### 3.2.3. Topical Phage Therapy for Secondarily Infected Burn-Mediated Ulcers 

A case report describes the successful decolonization of *Pseudomonas aeruginosa* (*PsA*) from the burn surface using topical phage potentiating subsequent skin grafting 3-days later. The *PsA* targeting topical phages were applied via impregnation of filter paper discs (10^3^ pfu) once to the burned surface. The authors noted a rise in phage concentration in the discs suggesting efficacious phage-mediated bacterial killing [18]. Subsequently, the phage was applied to the entire burn surface resulting in negative cultures 3 days later potentiating successful grafting [18].

Thirty patients suffering from secondarily (MDR *PsA*) infected burns, failing antibiotic therapy experienced variable efficacy employing adjunctive topical phage therapy in clinical wound healing and facilitation of skin grafting. Bandages soaked with 10^10^ phages/mL were applied three times daily for 5–17 days. The authors suggested clinical improvement in 15 patients (minimal wound discharge), slight improvement in 9 cases, good to excellent skin graft purchase in 18 patients, and “sterile post-phage treatment cultures” observed in 12 patients [19].

In a contemporary safety evaluation (executed in 2007–2008), a fixed 3-phage cocktail (targeting *PsA and S. aureus*) called BFC-1 (whose composition was selected to target the bacterial strains representing the burn unit microbiome) was safely applied to 9 colonized burn patients (via a single “spray” application) at a dose of 10^7^ phages/cm^2^. The follow-up evaluation was performed between 2–5 h post-application to assess safety (confirmed), and acquire a punch biopsy for culture. There was no difference in the microbiological bacterial burden of the wound from pre to post-biopsy sampling. There were no inferences rendered regarding efficacy [20]. The authors noted the inferiority of exploiting spray delivery given the unavoidable drainage from the wound bed. 

In a randomized phase 1/2 trial (NCT02116010), 27 patients >18 years of age exhibiting a confirmed burn-mediated wound infection (microbiologically confirmed due to *PsA*) were recruited from nine burn centers in hospitals in France and Belgium between 22 July 2015 and 2 Jan 2017. Patients were randomized to a cocktail of 12 lytic anti-*PsA* bacteriophages (PP1131; 1 × 10^6^ plaque-forming units [PFU] per mL) applied via an impregnated alginate dressing (*n* = 13), or standard of care (SOC) (1% sulfadiazine silver emulsion cream) (*n* = 13). Both treatments were administered as a daily topical treatment for 7 days, with 14 days of follow-up. Of import, only 12 patients received the phage therapy at day 0, 10 completed 7 days of treatment and 9 completed 14 days of follow-up. The intervention group was older, and exhibited a higher bacterial burden at day 0 than that of the SOC group. The primary endpoint involved sustained reduction in bacterial burden (by at least 2 quadrants via a 4-quadrant method) and was significantly improved with SOC therapy [47 h (23–122)] versus [144 h (95% CI: 48 h-not reached)] in the PP1131 group (hazard ratio 0·29, 95% CI 0.10–0.79; *p* = 0.018). Ultimately, although progress was delayed in the phage group, clinically germane reductions in bacterial burden was observed accompanied by fewer adverse effects (compared to SOC). The authors note that the bacterial burden coupled to the vastly lower than projected phage titers in the treatment group (1 × 10^2^ PFU/mL) yielded a low phage to bacteria multiplicity of infection (MOI), and may have encountered trapping within the alginate matrix template applied to the wound likely contributing to the observed inferior efficacy, and evolving bacterial phage resistance [21]. Finally, of import, the bacterial-phage susceptibility at day 0 significantly predicted reaching the primary endpoint (*p* < 0.0001) supporting implementing a personalized phage approach to treatment.

A sub-cohort of 49 patients afflicted with burn-mediated pyogenic infections (encompassing *S. aureus*, and *E. coli*, *Klebsiella*, *Proteus*, and *Pseudomonas* spp.) was described within a review of treatment results from 1307 MDR suppurative infections spanning a host of clinical syndromes between the years 1987–1999. Most of these patients failed antecedent antibiotic therapy, and were subsequently treated with targeted phage therapy derived from the Institute of Immunology and Experimental Therapy, Wroclaw, Poland. Targeted phage therapy implied isolation of the bacterial strain(s) from patient specimens, and determination of phages exhibiting active killing in vitro prior to preparation of sterile lysates for therapy. The phage treatment was administered topically (further details regarding dosage, frequency, and duration unavailable) as well as orally (three times daily (TID) prior to meals and following gastric neutralization). Duration of therapy averaged 32 days for the entire cohort. Forty-two patients experienced full recovery (clinical and microbiological), while the balance experienced clinical improvement (albeit with detectable bacteria) [22].

#### 3.2.4. Topical Phage Therapy in the Treatment of Infected Diabetic Foot (Toe) Ulcers

A contemporaneous case series (six patients) was recently published describing the efficacy of weekly topical administration of a fully sequenced *S. aureus* targeting phage “Sb-1” (exhibiting very broad activity against a high percentage of circulating *S. aureus* strains) in achieving healing of complicated infected diabetic toe ulcers (proven colonized with *S. aureus*). Many of these ulcers were associated with underlying osteomyelitis, and were refractory to antecedent antimicrobial therapy and optimal revascularization procedures. Therefore, the topical phage delivery was employed as salvage therapy as many of these cases were otherwise resigned to amputation as the remaining viable treatment [23,24,25]. The microbiological data acquired from wound cultures yielded one case positive for methicillin-resistant *S. aureus* (MRSA), with the balance of cultures yielding methicillin sensitive *S. aureus* (MSSA). For experimental treatment, the phage preparation was dripped into the wound cavity, followed by gauze packing which was placed over the wound. The gauze packing was soaked (impregnated) with the phage preparation [0.1 to 0.5cc of phages (10^7^ to 10^8^ pfu/mL), depending on ulcer volume], and then covered with petroleum (Xeroform) gauze preventing phage solution from being wicked away from wound, and finally wrapped with dry gauze. The patient was instructed to leave the treatment in place for 48 h before removing, and thereafter replacing the treatment with moist dressings. This protocol was repeated weekly until the ulcer became too small to pack [23,24,25]. All ulcers exhibited healing after an average 7 weeks of therapy, accompanied by swift resolution of inflammation, and a smooth continuous improvement in healing. 

#### 3.2.5. Topical Phage Therapy in Undisclosed (Uncharacterized) Chronically Infected Wounds 

In a contemporary investigation, 20 patients (aged 20–60 years of age) experiencing chronic refractorily infected non-healing ulcers/wounds (failing (SOC) therapy including optimal surgical debridement and antibiotics) were administered salvage topical phage therapy (from Jan 2015 to June 2016). The phages were confirmed to target the infecting bacterial isolates which encompassed (*E. coli*, *S. aureus*, or *PsA*). The topical phage cocktails were delivered on alternating days for up to 10 days, in all cases achieving sterile wounds and evidence of incipient wound healing. Seven patients achieved complete healing by day-21 of follow-up while in others healthy margins and healthy granulation tissue were observed [26].

Topical phage therapy successfully treated 16 of 31 patients experiencing chronic suppurative cutaneous infections (heterogeneous in classification) due to varied pathogens (*Pseudomonas, Staphylococcus, Klebsiella., Proteus, and Escherichia* spp.) failing antecedent SOC therapy including antibiotics [27]. There were 21 mono-infections; however, 10 infections were polymicrobial. The phages were confirmed to target (lyse) the patient’s bacterial isolate in vitro. Antibiotics were discontinued during phage therapy but rheologic agents allowed. Topical phages were administered TID for a varying period of 2–16 weeks of treatment. Additionally, phages were prescribed orally 3–4 times daily prior to meals and after gastric neutralization. The authors noted overall clinical improvement accompanied by reduced local inflammation, microbiologic eradication, and expedited wound healing. Using a descriptive scale, 16 cases achieved outstanding results, while 7 and 2 patients exhibited marked and transient improvement, respectively. The microbiological results (endpoints involved “3 subsequent negative cultures”) mirrored the clinical results. Of note, adverse events thought attributed to topical phage administration included pain intensification in 2-patients and eczematous flares near the point of application in 4-patients [27].

A sub-cohort of 20 patients afflicted with cutaneous infections (furunculosis, decubitus ulcers and abscesses) was described within a review of treatment results from 138 “septic” infections spanning a host of clinical syndromes. Most of these patients within the entire cohort [125(90.6%)] experienced chronic, MDR infections, which failed antecedent antibiotic therapy. The targeted phage therapy employed was formulated in similar fashion as described above [22]. Of 138 cases, 129 (93.5%) achieved a good therapeutic result (manifest by control of the infection and healing of the local lesion). The phage treatment in both the entire cohort as well as the sub-cohort of cutaneous infections was administered primarily via dual modes of delivery, (a). topically—TID (further details regarding dosage, and duration unavailable), and ((b). orally TID (prior to meals and following gastric neutralization). An outstanding result manifest by complete recovery was achieved in 5 patients, with 14 patients achieving complete healing (accompanied by “liquidation of the suppurative process”). Only one adverse event, an allergic reaction was reported from local wound application [28].

A total of 48 study subjects experiencing recalcitrant wounds (27 diabetic) were administered personalized topical phages (some poly-microbial wounds receiving polyvalent bacterial targeting phages) applied daily, on an alternate day for 5 to 7 treatments between Aug 2018 and May 2019. At 3-months, microbiological eradication of the original infecting isolate was observed in 48/48 (100%), and complete healing achieved in 39/48 (81%) of patients [29].

## 4. Discussion

Interpretation of the Safety and Clinical Efficacy Employing Topical Phage Therapy in Chronically Infected Wounds

Wound healing involves a complex process of regulated, coordinated, and sequential activity of multiple interconnected pathways. A host of novel compounds are under scrutiny, which may promote an optimal balance of pro and anti-inflammatory mediators and angiogenesis in the local wound milieu, thereby potentiating wound healing. Additionally, infectious eradication is paramount, and is becoming increasingly arduous given the advent of MDR bacteria [2,3,4,5,6,7,8,9,10,11,12,13,14]. In this systematic literature search we identified two clinical studies reporting upon results from randomized trials, one of which was a pure safety evaluation, while the other sought to assess reductions in microbiological burden [16,21]. Neither trial was designed to assess clinical parameters including complete wound healing. The balance of studies identified, satisfying the inclusion criteria involved a case study, case series, and observational studies. We included two studies reporting observational data for a heterogeneous class of cutaneous wounds, which was extracted from observational data reporting upon a spectrum of clinical syndromes associated with suppurative infections [22,28]. We believe this data was important to integrate within our selected articles as it may reflect the overall uncontrolled clinical outcome data derived from the institutes in Wroclaw, Poland, and Tbilisi, Georgia, both pioneers exhibiting an established history in use of adjunctive clinical phage therapy. Furthermore, while not meeting strict (or even relaxed) inclusion criteria, we acknowledge numerous book chapters, review articles, and abstracts which although heterogeneous in reporting, provide signals of safety and efficacy of topical (and alternative routes of phage delivery) in wound therapy (microbiological eradication and wound healing).

Given the patent heterogeneity in all aspects of the observational studies identified [enrollment criteria, demographics, wound classifications, wound microbiology, antecedent chronicity and prior wound treatments employed, concomitant and uncontrolled, antibiotic usage, phage formulation employed, phage regimen used (dosage, route [topical, or topical + oral], frequency and duration of administration), and predominantly uncontrolled observational data culled from small subject numbers], we are unable to pursue formal statistical analyses to address clinical outcomes. Rather, we elect to present important descriptive analyses as likely more informative.

The studies most saliently confirm the safety in exploiting phage therapy (whether administered topically or orally). Notably, we did identify a few adverse events considered possibly attributable to topical phage delivery including an isolated potential allergic reaction, and increased localized pain, and eczematous changes (2 and 4 patients respectively). Although predominantly observational data, and with many of the studies assigning subjective descriptive scales establishing the germane clinical outcome of wound healing, there appears to be a significantly robust signal of efficacy using adjunctive topical phage therapy. Partial or complete wound healing was achieved in 242/273 (89%) patients. Complete wound healing, wound epithelialization, or improvement sufficient to potentiate grafting was achieved as desired outcomes in 225/321 (70%) of patients. Microbiological eradication rates mirrored the clinical efficacy results. Despite acknowledging the limitations from the observational and uncontrolled nature of most studies, and the profound heterogeneity observed, these were a highly selected cohort of patients, exhibiting chronic refractory wound infections, who had failed prior SOC therapies, including antecedent antibiotic treatment(s). Furthermore, many studies acknowledged the importance of using personalized phages confirmed to target (kill or lyse) the acquired patient bacterial isolate in vitro. Additionally, efficacy appears to have been undermined with indirect retrospective conformational data suggesting that the phages either failed to target the infecting bacterial isolate, or was administered at insufficient concentrations [inferior multiplicities of infection (MOI)] [21]. Two recent seminal reports included within this review provide a most exciting testimony to the potential clinical utility of adjunctive topical phage therapy [17,24,25]. Both studies provide nearly unequivocal evidence that topical phage therapy contributed to complete clinical wound healing in patients refractory to antecedent antibiotic therapy. Furthermore, in the latter study, although results are observational and confined to assessing outcomes relative to the patient’s historical control, it appears phage therapy circumvented salvage therapies including amputation. Additionally, this study fosters speculation that superior outcomes may be achieved with extended courses of therapy, as complete wound healing was realized in many cases when employing protracted courses [24,25], and eradication of a dominant virulent pathogen (notably *S. aureus*) may be sufficient to promote healing in presumptively poly-microbially infected diabetic wounds.

Therefore, we conclude that topical phage therapy is safe, and does indeed exhibit efficacy signals in complicated cutaneous infections warranting further evaluation via well designed clinical investigations. We posit personalized “targeted” phages confirmed to lyse the patient’s isolate in vitro may be best employed for clinical therapy, but acknowledge that well designed and characterized fixed cocktails, may exhibit sufficient targeted lytic activity against the majority of circulating bacterial strains to warrant their initial clinical application awaiting confirmation of in vitro lytic activity. 

## 5. Preparations for a Randomized Clinical Trial Evaluating the Safety and Therapeutic Efficacy of Adjunctive Phage Therapy in Infected Diabetic Foot Ulcers

### 5.1. Background

#### 5.1.1. Diabetic Foot Infections (DFI): A Canonical Paradigm and Model for Complicated Refractory Infected Cutaneous Wounds/Ulcers

Diabetic foot ulcer infections (DFI) are a common complication of diabetes and represent a major public health issue given the epidemiology and increasing refractoriness to treatment stemming from MDR bacteria. These wounds often require multiple surgical interventions (debridement), ultimately funneling to amputations as definitive salvage therapy. Antibiotic treatment of these complicated infected ulcers is undermined by the polymicrobial nature of the infection, escalating MDR, poor tissue vascularization, immune depression (dysregulation and incoordination), formation of microbial biofilms and patient co-morbidities [24,25,29]. In addition to all the purported advantages of phage as adjunctive treatment for bacterial infections discussed in the background, topically delivered phages may (1) penetrate (via diffusion) deeply into infected tissue despite poor vascularization, and (2) degrade the local biofilm [24,25,29]. We assert that DFI represents the canonical paradigm of complicated refractory infected ulcers. Therefore, therapeutic phage efficacy in DFI likely translates to most complex chronically infected wounds.

#### 5.1.2. Protocol Overview: Phage Therapy for Treatment of Chronic Diabetic Foot Infections (DFI)

We will elaborate further upon the preparations and infrastructure required to pursue the clinical trial, awaiting formal publication of the clinical trial design until after study initiation. To our knowledge this would be the first formal randomized clinical trial employing an efficient adaptive design, investigating adjunctive personalized phage therapy administered via both IV and topical routes to treat refractory DFI. As discussed earlier, intuitively, we suspect concomitant phage delivery via both modalities may be necessary to expedite infectious resolution. Topical administration may gain superior access to the bacterial burden, optimize biofilm degradation, and solicit a salubrious local immunological milieu, while IV administration may eradicate the sequestered infection in the deep tissue recesses less available to access via the topical route, and treat concomitant systemic infection.

We will exploit precision-based personalized (“targeted”) phage therapy. This involves acquiring bacterial isolates from all patients considered for phage therapy, and then formulating personalized phage cocktails (mixtures) which are confirmed to exhibit in vitro lytic activity. This personalized approach potentiates an iterative cycle of novel targeted phage acquisition if required [i.e., encountering bacterial resistance to initial phage therapy]. We believe this approach is superior to using fixed phage cocktails (regardless of engineering undertaken) as the latter approach may ostensibly lack therapeutic efficacy if not lytic against the infecting bacterial isolate, while disallowing adaptability to the inexorable evolving bacterial resistance emanating from the selective pressures imposed upon the predating phages.

The enormous bacterial genetic diversity mandates a vast library of phages from which to screen and draw upon to confirm appropriate phage-bacterial targeting. Within this phage library, all representative phages are meticulously characterized for specificity in bacterial killing, and absence of carriage of virulence and antibiotic resistance factors. We refer to our “library” of phages as the PhageBank^TM^, which is continually expanded to ensure sufficient coverage of the circulating infectious bacterial isolates, and to facilitate expedited isolation of personalized (targeted) phage cocktails in real time to accommodate evolving resistance during therapy. Prior to study initiation, we will have acquired the epidemiology of circulating pathogens culpable for diabetic foot infections at each enrolling site. We will acquire a representative sampling of bacterial isolates from each participating institution and ensure our PhageBank^TM^ houses sufficient phage diversity to target >80% of these institutional centric circulating isolates prior to study initiation. 

**Figure 2:**
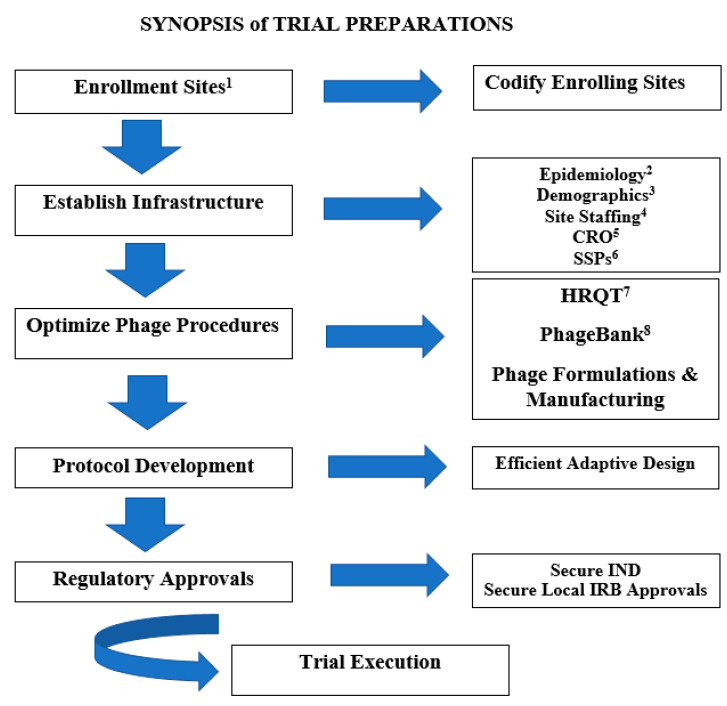
Synopsis of trial preparations. 1. This is intended as a multi-site study. 2. Epidemiology: Acquire epidemiology of the circulating bacterial isolates (including incidence, and their virulence and antimicrobial susceptibility profiles) culpable in chronic diabetic ulcer infections at each site. Additionally, screen the PhageBankTM for phage targeting of a representative sampling of the circulating bacterial isolates at each site, and ensure >80% coverage prior to study initiation. Finally, confirm standard of care including antibiotic selection practices. 3. Demographics stratified by each participating site. 4. Site Staffing: Clinical Research Nurse, Research Associates, Research Physicians, Laboratory (including Microbiological) Support, Pharmacy Support, Data Management Support. 5. CRO: Clinical Research Organization. The contracted research organization (CRO) will be contracted by sponsor. The CRO will oversee study execution (provide oversight), and will perform (site initiation visits, surveillance clinical monitoring visits, confirmation of appropriate site staffing and support, liaison services between study sites and the sponsor, execution of study specific procedures (SSPs), and centralized data management). 6. SSPs: Study Specific Procedures. The SSPs will include (but is not limited to) procedures delineating (a). Staffing, ((b). Microbiological Sampling, (c). Phage Administration Procedures, (d). Storage, (e). Shipping, (f). Regulatory Communications, (g). Data Entry. 7. HRQT: Host Range Quick Test (see narrative). 8. PhageBankTM: Ensure >80% phage coverage targeting known circulating bacterial isolates culpable in diabetic foot ulcer (DFU) infections.

Finally, our screening approach to confirm the phage-bacterial killing activity uses the proprietary Host Range Quick Test (HRQT) to be elaborated upon below. Figure 2 provides a flow chart schematic of the trial execution. We will next provide a detailed exposition of the HRQT and the PhageBank^TM^ both unique and fundamental supporting procedures potentiating this investigation.

### 5.2. The HRQT Assay

**Figure 3:**
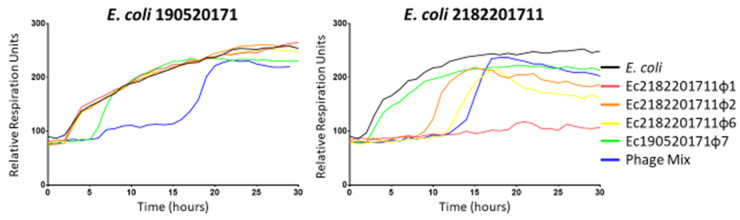
MCR-1 expressing *E. coli* strains were grown in TS broth (black). *E. coli* strains were grown with a MOI 10 of phage Ec2182201711φ1 (red), Ec2182201711φ2 (orange), Ec2182201711φ6 (yellow), Ec190520171φ7 (green), or a mixture of all phage together (blue).

The HRQT assay is an automated assay that measures bacterial cellular respiration using a tetrazolium dye (a colorimetric assay) [30]. During active bacterial growth, the increased cellular respiration reduces tetrazolium dye and produces a color change. The HRQT assay is run in 96-well plates that are robotically loaded with 10^4^ bacterial cells per well, tetrazolium dye, and varying concentrations of phage. Plates are then read every 15 min for several hours in an OmniLog instrument (Biolog Inc.) and “kill curves” are generated (see Figure 3). The y-axis reveals the relative respiration units and the x-axis displays time. Bacterial growth suppression is indicated by the isolated bacterial curve remaining flat for an extended period. Within this HRQT platform, individual and multiple phages (phage cocktails or mixtures) may be assessed for synergy (or frank antagonism) in targeting the bacterial isolate. Efficacious phage(s) suppress bacterial proliferation for a sufficient period reflecting in vitro (and in our experience by extension) in vivo efficacy.

For example, in Figure 3 we have two bacterial strains *E. coli 190520171* (left panel), and *E. coli 2182201711* (right panel) depicted which were assayed in the HRQT platform (black curves) at approximately 10^4^ cfu (colony forming units). To these wells in our HRQT was added 4 unique bacteriophages (all at a MOI of 10 or approximately 10^5^ pfu): Ec2182201711φ1 (red), Ec2182201711φ2 (orange), Ec2182201711φ6 (yellow), Ec190520171φ7 (green), or a mixture of all phage together (blue). For the former bacterial strain *E. coli 190520171* (left panel), we observe only one phage (Ec190520171φ7 (green)) which exhibited efficacy in delaying bacterial growth eclipsing 4 h (considered a surrogate for in vivo phage-mediated clinical therapeutic efficacy). For the latter bacterial strain *E. coli 2182201711* (right panel), three phages [Ec2182201711φ1 (red), Ec2182201711φ2 (orange), and Ec2182201711φ6 (yellow)] delayed bacterial growth. We observe synergy (the delayed growth observed exceeding the sum of the delay observed upon adding individual constituents of a mixture) in delaying bacterial growth when subjecting the bacterial isolate (left panel) to all 4 phages (comprising a phage cocktail). For the bacterial isolate *E. coli 2182201711* (right panel), we observe antagonism when employing the phage cocktail relative to the isolated potency of the individual phage Ec2182201711φ1 (red). Clinically (and intuitively) we wish to use phage cocktails to improve potency against the targeted infection, and concomitantly reduce the risk of developing bacterial resistance to the phage during treatment. Therefore, this brief exposition underscores the need to assess each phage individually, as well as their combination when envisioning employing a phage cocktail (mixture) for clinical practice. 

**Figure 4:**
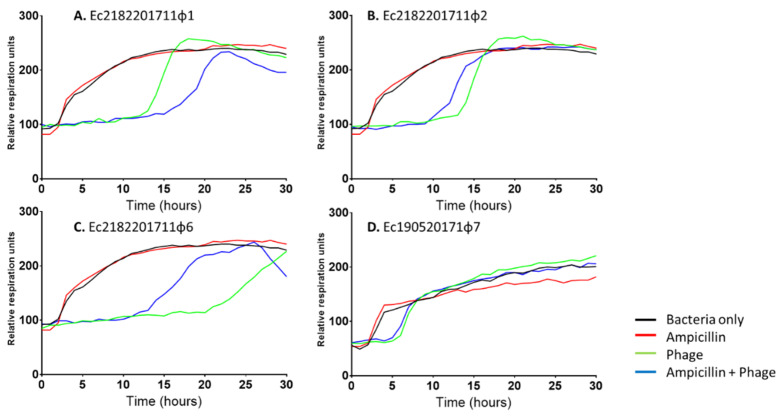
MCR-1 expressing *E. coli* strains were grown in only TS broth (black), with ampicillin (32 µg/mL) (red), with their respective phage (green), and a combination of both ampicillin and their phage (blue). Panels are grown with *E. coli* 2182201711 and (A) Ec2182201711φ1, ((b) Ec2182201711φ2, (C) Ec2182201711φ6, or (D) *E. coli* 190520171 with Ec190520171φ7. The MIC for each isolate was 16 µg/mL to ampicillin.

In addition to exploiting the HRQT to assess synergy in polyvalent phage cocktails, we may also assess in vitro phage-antibiotic synergy (or antagonism). As an illustrative example, in Figure 4, panels A, B, and C depict *E. coli* 2182201711 grown with an individual phage; Ec2182201711φ1 (panel-A), Ec2182201711φ2 (panel-(b), and Ec2182201711φ6 (panel-C) or the antibiotic ampicillin (32 µg/mL). In all panels, we note antibiotic resistance. However, the combination of each individual phage and ampicillin revealed; synergy (panel-A), mild antagonism (panel-(b), and frank antagonism (panel-C). In panel D, we depict *E. coli* 190520171 grown with the phage Ec190520171φ7 and ampicillin. We do not observe any synergy in this phage-antibiotic combination. From this brief exposition, we reveal that the HRQT may be exploited ideally as a rapidly deployable, portable, cost-effective clinical diagnostic device to identify optimal phage-phage and phage-antibiotic synergy (and avoidance of potential in vivo antagonism) for dictating phage formulations to use in clinical applications. 

### 5.3. PhageBank^TM^

The PhageBank^TM^ has evolved into a large and dynamically growing collection of purified phages that lyse clinically relevant MDR pathogens including *Staphylococcus aureus*, *Escherichia coli*, *Klebsiella pneumoniae*, *Acinetobacter baumannii*, *Pseudomonas aeruginosa*, and *vancomycin-resistant enterococci*. All phages included in PhageBank^TM^ are purified, sequenced, and determined to be devoid of deleterious genes including antibiotic resistance genes and virulence determinants. Phages found to contain toxin genes or other potentially detrimental genomic elements are discarded [31]. Phages in PhageBank™ are manufactured, purified, assayed for endotoxin and host cell proteins, aliquoted into single-dose vials, and tested for sterility. The vials are stored at −80 °C at which temperature they appear to be stable indefinitely. We envision perching the PhageBank^TM^ locally (co-located with the HRQT) to facilitate expedited identification, formulation, and expedited administration of phage cocktails for clinical applications.

### 5.4. Exploratory Insights to Optimizing Adjunctive Phage Therapy

As phage therapy resides within its clinical infancy in Western nations, we will design the clinical trial ensuring pursuit of greater insight into numerous important aspects of clinical phage therapeutics including: (1) phage pharmacokinetics, and pharmacodynamics; (2) optimization of therapeutic regimens clarifying dose, and route, frequency, and duration of administration; (3) optimization in the relative dosing, and sequence (“staggering”) of administering antibiotics and phage, [32,33,34,35]; (4) synergy with adjunctive wound therapeutics (biofilm degrading agents); (5) clarification of the depth and breadth of the host immune response (innate and adaptive) solicited, and influence upon treatment efficacy; (6) optimization of HRQT procedures for identifying phages fostering intrinsic biofilm degradative activity; (7) innovations to minimize bacterial resistance during treatment, and (8) optimization of topical phage delivery maximizing potency, stability and durability, kinetics (controlled release), penetration, and longevity. Saliently, for the final assessment, we acknowledge requirements to optimize the phage delivery vehicle (formulations) [direct phage instillation onto the wound bed, impregnated into standard or novel clinical dressings, or use of novel encapsulation methods, i.e., hydrogels]. As an illustrative example, impregnated hydrogels with phage and antibiotics improved and expedited microbiological eradication and wound healing in murine models [36]. Optimizing the phage release kinetics and the dressing platform engenders secondary benefits including delays in requisite wound manipulations and dressing changes, protection from mechanical injury and secondary bacterial wound colonization, mitigation of heat and moisture loss from injured tissues, optimization of exudate release, wound oxygenation and moisture (vapor) pressures, and promotion and entrainment of growth factors and immunological mediators. We envision integration of novel formulations in streamlined fashion as the protocol is executed (as insight is derived from concurrent pre-clinical investigations) exploiting a flexible, adaptive clinical design.

## 6. Conclusions

This systematic literature review identified a heterogeneous compilation of predominantly observational data reporting upon clinical applications of topical phage therapy. The review most saliently confirms the safety in exploiting phage therapy (whether administered topically or orally). Although predominantly observational data, there appears to be a significantly robust signal of efficacy using adjunctive topical phage therapy. Partial or complete wound healing was achieved in 242/273 (89%) patients. Microbiological eradication rates mirrored the clinical efficacy results. Despite acknowledging the limitations from the observational nature of most studies, these were a highly selected cohort of patients who had failed prior SOC therapies, including antecedent antibiotic treatment(s). Topical phage therapy represents a safe and promising and potentially transformative treatment for recalcitrant infectious cutaneous wounds and warrants concerted research given the advent of MDR and lack of novel antibiotics in our armamentarium. 

## Figures and Tables

**Table 1:** Delineation of data extraction from included articles.

Author	Year Published	Wound Classification	Microbiology	Phage Characterization	Patient-#	Phage ^&&^ Regimen (a). Dose ((b). Frequency (c). Duration	Follow-up	Efficacy Results Reported ^&&^ (a). Clinical Healing (b). Microbiological Eradication (c). Safety Evaluation
(1). Slopek	1983	Skin infections (furunculosis, abscesses, and decubitus ulcers)	Numerous virulent infections	Unknown, but phages were personalized and targeted patient’s bacterial isolate in vitro. Additionally, phages administered orally	20	(a). Unk (b). Topically: TID Orally: TID (c). Unk	Unk^1^	(a). An outstanding result manifest by complete recovery was achieved in 5 patients --14 patients achieving complete healing (accompanied by “liquidation of the suppurative process”). (b). Microbiological results mirror the clinical (c). Safety established overall. One adverse event, an allergic reaction was reported from local wound application
(2). Cislo	1987	Purulent Ulcers	*Staphylococcus*, *Pseudomonas*, *Klebsiella*, *Proteus* spp. and *E. coli*	Unknown, but phages were personalized and targeted patient’s bacterial isolate in vitro. Additionally, phages administered orally	31	(a). Unk ^1^ Dose (b). TID-QID ^2^ (c). 2–16 weeks	2–16 weeks	(a). Outstanding therapeutic effect (wound healing) in 16 patients; Marked and transitory clinical improvement in 7 and 2 patients, respectively. (b). Microbiological results mirror the clinical (c). Safety established overall. Potential attributable adverse events ascribed to phage therapy included intensified local pain and eczematous changes.
(3). Abul-Hassan	1990	Burn-Mediated	*Pseudomonas aeruginosa (PsA)*	Unknown	30	(a). 10^10^ pfu/mL ^3^ (Impregnated Gauze) (b). TID (c). 5–17 days	5–17 days	(a). Improvement in 15, slight improvement in 9, and no improvement in 6 patients respectively --Graft purchase in 18/30 patients (b). Sterile cultures in 12/30 patients (c). Safety established
(4). Weber-Dabrowska	2000	Burn-Mediated	*S. aureus*, and *E. coli*, *Klebsiella*, *Proteus*, and *Pseudomonas* spp.	Unknown, but phages were personalized and targeted patient’s bacterial isolate in vitro. Additionally, phages administered orally	49	(a). Unk (b). Unk topical Oral delivery TID (c). Unk	Unk	(a). Clinical resolution in 42/49 (86%) Improvement in the balance (7 patients) (b). Microbiological clearance in 42/49 (86%) Reduced bacterial burden in the balance (c). Safety established
(5). Markoishvilli	2002	Venous Stasis and uncharacterized “ulcers/wounds”	*E. coli* *Proteus* spp. *Pseudomonas* spp. *Staphylococcus* spp.	PhagoBioDerm Phages (10^6^ pfu/cm^2^) targeting [*PsA*, *E. coli*, *(S. aureus)*, *Streptococcus*, and *Proteus* spp.]	96	(a). # Dressings applied varied (based on wound size) (b). Reapplied PRN ^4^ (typically 3–7 days) (c). N/A	6 days–15 months	(a). Clinical efficacy in 67/96 (70%) patients. Improvement (reduced ulcer size and elimination of purulent drainage in another 24 patients. (b). All 22 patients for whom microbiological data had been collected were in the completely healed group. (c). Safety established
(6). Jikia	2005	Radiation Injury	*S. aureus*	PhagoBioDerm The *S. aureus* isolates were susceptible to the phage preparation in this product	2	(a). # Dressings applied varied (based on wound size) (b). Reapplied PRN (c). N/A	7 days	(a). Clinical wound healing in 2/2 (100%) subjects, both failing antecedent antibiotics (b). Microbiological eradication in 2/2 (100%) (c). Safety established
(7). Marza	2006	Burn-mediated	*PsA*	Derived by Dr. Soothill	1	(a). ~2 × 10^3^ pfu Applied via 2 sterile paper discs (25 mm), then application to entire surface (b). × 2 doses (c). Unk	3 days	(a). Clinical wound healing (partial) (b). Infectious eradication of *PsA* Facilitating a successful graft (c). Safety established
(8). Rhoades	2009	Venous Stasis Ulcers (infected or uninfected)	N/A^5^ [Wounds were not cultured to assess susceptibility to the phage constituents]	8-phage cocktail (10^9^ pfu/mL per phage) targeting *Pseudomonas* spp., *E. coli* spp., and *S. aureus* *“*WPP-201”	39 (18 received phages)	(a). 4 mL Impregnated Dressing (b). Weekly (c). 12-weeks	24 weeks	(a). Wound epithelialization achieved in 17/21 control patients and 12/18 treated patients (non-significant) (b). Microbiological Outcomes: N/A ^5^ (a phase I safety evaluation) (c). Safety established
(9). Rose	2014	Burn-mediated	MDR *PsA or S. aureus*	3-phage cocktail targeting *PsA* and *S. aureus “BFC-1”* *Please note that this cocktail was active against the strain populating the burn wound center*	9 patients (10 burn applications)	(a). 10^7^ phages/cm^2^ (average dose) (b). × 1 dose (c). × 1 dose	2 to 5 h	(a)/(b). Microbiological and Clinical Outcomes: [No change in the microbiological (bacterial) load from pre to post-biopsy wound sampling] (c). Safety established
(10). Fish	2018	Diabetic toe ulcers	*S. aureus*	*S. aureus* targeting phage “Sb-1”	6	(a). 10^7^ to 10^8^ pfu/mL Impregnated Dressings (b). Weekly (c). Variable	7 week median	(a). Clinical wound healing in 6/6 (100%) patients (b). Microbiological data N/A (c). Safety established
(11). Jault	2019	Burn-mediated	*PsA*	12-phage cocktail targeting *PsA* “PP1131”	12-treated 13-placebo “efficacy population”	(a). 10^2^ pfu/mL (b). Daily (c). 7 days	21 days (14 days follow-up)	(a). Clinical Healing: N/A (b). Microbiological Endpoint: Reduced bacterial burden HR ^6^: 0.29, 95% CI ^7^ 0.10–0.79; *p* = 0.018 favoring SOC ^9^ (1% sulfadiazine silver emulsion cream) for microbial burden (c). Safety established
(12). Gupta	2019	Purulent ulcers	*E. coli*, *S. aureus*, or *PsA*	Unknown (however, a personalized 3-phage cocktail was identified to target the organism in all cases).	20	(a). 10^9^ pfu/mL (total pfu dependent upon wound area) (b). QOD ^8^ (c). 6–10 days	3-months	(a). Clinical healing in 7/20 (35%) patients -20/20 patients experienced improvement (b). Microbiological sterility achieved in 20/20 (100%) patients (c). Safety established
(13). Patel	2019	Heterogeneous Diabetic ulcers (>50%)	Bacteria recovered at >10% E. coli (37.5%) PsA (31%) S. aureus (31%) Klebsiella pnuemonia (12.5%)	A personalized phage cocktail was identified to target the organism in all cases. Some were polyvalent targeting the multivalent bacterial wound infection.	48	(a). 500 µL/cm^2^ (10^9^ pfu/mL) (b). QOD (c). 5 to 7 treatments	3-months	(a). 39/48 (81%) cure (b). Microbiological eradication established in 48/48 (100%) (c). Safety established Increased lymphocytes observed

^1^ Unk: Unknown; ^2^ TID: Three times daily; QID: Four times daily; ^3^ pfu: plaque-forming units; ^4^ PRN: As needed; ^5^ N/A: Not applicable; ^6^ HR: Hazard Ratio; ^7^ CI: Confidence Interval. ^8^ QOD: Every other day; ^9^ SOC: Standard of Care; ^&&^ Data presented only if provided. Many reports failed to report all potential parameters.

## Copyright Statement

I am a military Service member (CD) or an employee of the U.S. Government (B(b). This work was prepared as part of my official duties. Title 17 U.S.C. §105 provides that copyright protection under this title is not available for any work of the U.S. Government. Title 17 U.S.C. §101 defines a U.S. Government work as a work prepared by a military Service member or employee of the U.S. Government as part of that person’s official duties.
